# The Effect of Subcutaneous Fat on Electrical Impedance Myography: Electrode Configuration and Multi-Frequency Analyses

**DOI:** 10.1371/journal.pone.0156154

**Published:** 2016-05-26

**Authors:** Le Li, Xiaoyan Li, Huijing Hu, Henry Shin, Ping Zhou

**Affiliations:** 1 Department of Rehabilitation Medicine, The First Affiliated Hospital, Sun Yat-sen University, Guangzhou, Guangdong, China; 2 Guangdong Provincial Work Injury Rehabilitation Center, Guangzhou, Guangdong, China; 3 Department of Physical Medicine and Rehabilitation, University of Texas Health Science Center at Houston, Houston, Texas, United States of America; 4 TIRR Memorial Hermann Research Center, Houston, Texas, United States of America; Shanghai Jiao Tong University, CHINA

## Abstract

This study investigates the impact of the subcutaneous fat layer (SFL) thickness on localized electrical impedance myography (EIM), as well as the effects of different current electrodes, varying in distance and direction, on EIM output. Twenty-three healthy subjects underwent localized multi-frequency EIM on their biceps brachii muscles with a hand-held electrode array. The EIM measurements were recorded under three different configurations: wide (or outer) longitudinal configuration 6.8 cm, narrow (or inner) longitudinal configuration 4.5 cm, and narrow transverse configuration 4.5 cm. Ultrasound was applied to measure the SFL thickness. Coefficients of determination (R^2^) of three EIM variables (resistance, reactance, and phase) and SFL thickness were calculated. For the longitudinal configuration, the wide distance could reduce the effects of the subcutaneous fat when compared with the narrow distance, but a significant correlation still remained for all three EIM parameters. However, there was no significant correlation between SFL thickness and reactance in the transverse configuration (R^2^ = 0.0294, p = 0.434). Utilizing a ratio of 50kHz/100kHz phase was found to be able to help reduce the correlation with SFL thickness for all the three configurations. The findings indicate that the appropriate selection of the current electrode distance, direction and the multi-frequency phase ratio can reduce the impact of subcutaneous fat on EIM. These settings should be evaluated for future clinical studies using hand-held localized arrays to perform EIM.

## Introduction

Electrical impedance myography (EIM) is a noninvasive technology used in assessing muscle health by applying high frequency, very low amplitude current through the muscle and measuring the resulting voltage with sensing electrodes on the skin [1]. The underlying mechanism of EIM is that pathological changes in muscle produce disruptions in normal impedance characteristics [2]. Basic EIM parameters include (1) muscle resistance (R), representing the resistivity to current flow in the extracellular and intracellular fluids; (2) muscle reactance (X), indicating how the current flow is affected by cell membranes and by the various fascia of the body [3], and (3) phase (θ), which is defined as θ = arctan (X/R) [1].

EIM provides a quantification of muscle fiber size, structure and overall composition [1] and has been primarily used to evaluate muscle alterations in various neuromuscular diseases, such as amyotrophic lateral sclerosis (ALS) [4], spinal muscular atrophy (SMA) [5], Duchenne muscular dystrophy (DMD) [6,7], as well as in studies of normal aging [8,9]. In the early development of EIM technology, one of the basic forms was a linear EIM in which the adhesive current electrodes were set at a distant site from a series of voltage electrodes parallel to the axis of the muscle [10]. In order to promote the application of EIM in the clinic, a hand-held electrode array (HEA) was developed with relatively closer distances between the current and voltage electrodes [11–13]. Previous studies have shown that handheld electrode arrays have good repeatability and reliability for measuring muscle impedance [12].

When performing EIM, the electrodes are placed on the skin surface. The effect of the skin and subcutaneous fat under the electrodes on EIM variables has been a continued topic of discussion. Sung et al. found that for the medial gastrocnemius muscle in healthy subjects, there were significant correlations between the subcutaneous fat layer (SFL) thickness and the resistance and phase whereas the reactance was relatively not impacted by subcutaneous fat [13]. For the quadriceps muscle, Tarulli et al. showed that the SFL thickness did not contribute substantially to the phase [14]. Such discrepancies might be related to the different muscles and electrode configurations. Jafarpoor et al. applied a finite element model to simulate human upper arm muscles and demonstrated that increased SFL thickness resulted in a larger increase in resistance than reactance [15], and it was found that the electrode distance had dramatic contribution to the EIM outputs. More recently the impact of SFL on EIM was also investigated in diseased muscles. For example, a study with SMA children showed the EIM was related to SFL thickness although the association was much weaker than between EIM and the strength [5]. In addition, the frequency dependence of muscle evaluation has been exploited by emitting multi-frequency current to help extract various muscle-specific properties (i.e. anisotropy) [3]. Utilizing multi-frequency measures has also shown to be more sensitive in tracking disease progression [16]. Furthermore, taking the ratio of two different frequencies can improve the value of EIM by reducing the impact of SFL [7].

The objective of the current study was to assess the impact of SFL thickness on the localized muscle impedance of the biceps brachii muscle, as well as to observe the changes of the EIM data under different frequencies. We compared the effects of different electrode configurations on the EIM output and found that among the three EIM parameters, reactance is the least affected by the subcutaneous fat. Applying both a transverse current emitting configuration and a multi-frequency phase ratio can help reduce the impact of the subcutaneous fat. Therefore, these settings should be evaluated when applying EIM in future clinical studies.

## Methods

### Subjects

Twenty-three healthy subjects (11 female and 12 male, age 34.7±7.4 (mean ± standard deviation) years, ranged from 23 to 46 years) participated in this study. Subjects had no history of neuromuscular disease and had normal strength and bulk of biceps brachii. The study was approved by the Committee for the Protection of Human Subjects (CPHS) of University of Texas Health Science Center at Houston and TIRR Memorial Hermann Hospital (Houston, TX). All procedures were conducted according to the Declaration of Helsinki. All the subjects gave their written consent before experiment procedures.

### Experiment

**Fig 1:**
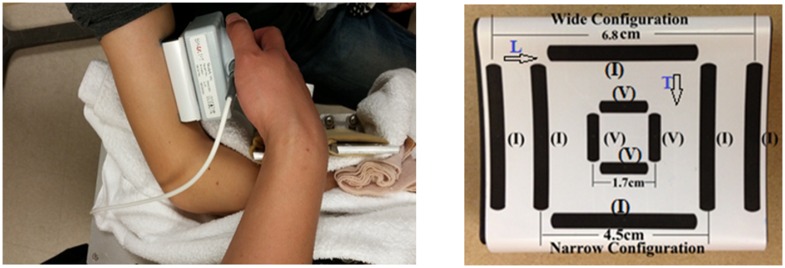
Position of the probe anpd a close up of the handheld electrode array configurations. (I) means current electrodes; (V) means voltage electrodes; L with arrow means the longitudinal direction; T with arrow means the transverse direction.

The experiments were performed on the biceps brachii muscle in the dominant limb of each subject (Fig 1). The subjects were seated in a height-adjustable chair with the examined arm put on a customized apparatus with elbow joint at 90° flexion and the shoulder at 45° abduction. The wrist joint was supported and placed in a neutral position. The laboratory maintained a constant temperature (approximately 22°C) during all the experiments.

**Fig 2:**
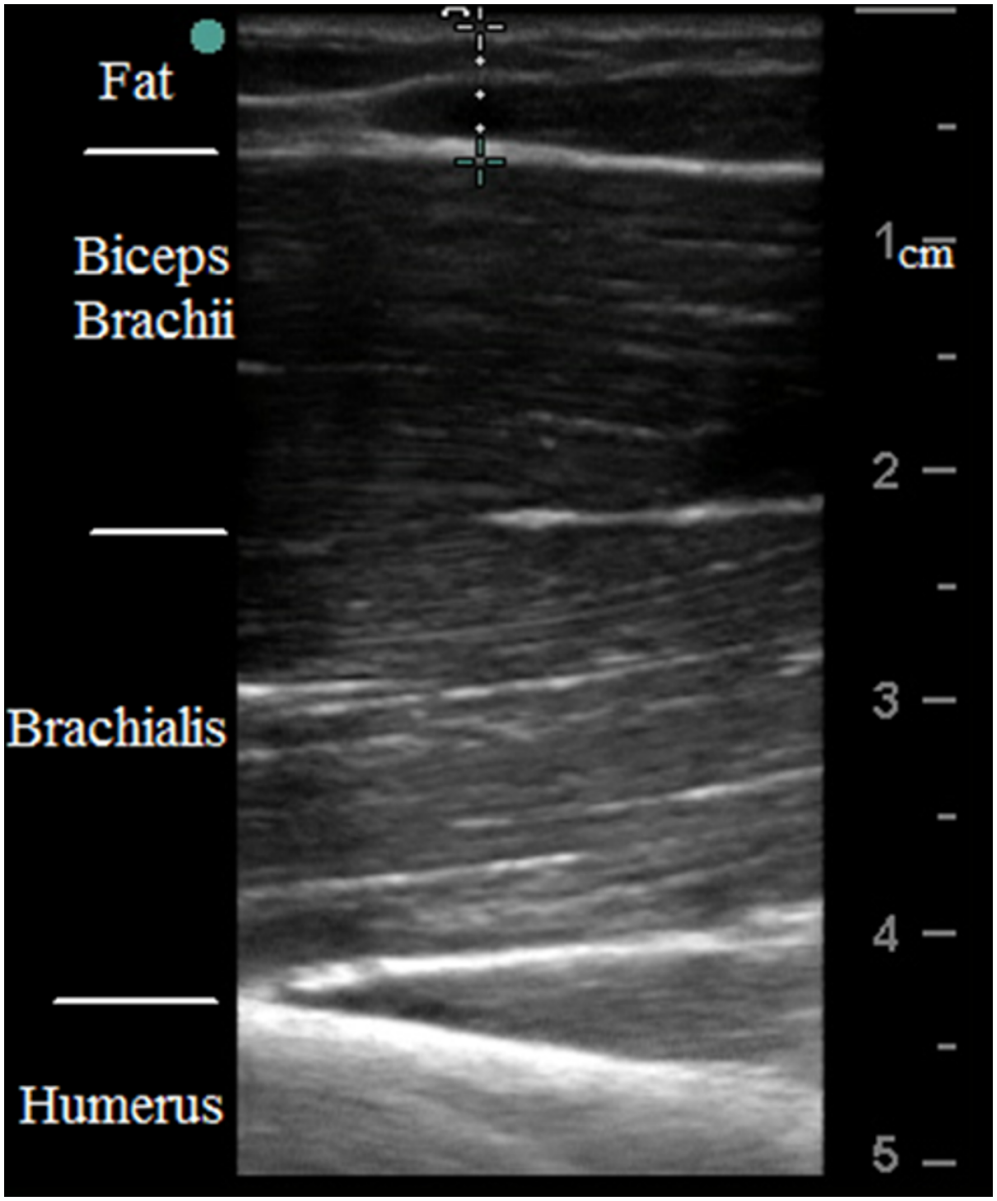
An example of ultrasound data showing the measurement of the SFL thickness.

Impedance measurements were performed using a handheld electrode array system (EIM1103, Skulpt Inc., MA) which sequentially applies a very low-intensity electrical current at frequencies ranging from 1 kHz to 10 MHz in discrete logarithmic steps, and measures the consequent surface voltages [12]. The sensor array (Fig 1) was placed longitudinally over the center of the muscle belly. The current-electrodes are designed in three configuration pairs. Two narrow (or inner) pairs have a separation distance of 4.5 cm (center to center) in both the longitudinal and transverse directions, and the wider (or outer) pair (only longitudinal direction) is separated by 6.8 cm. The voltage-measuring electrodes are the same distance for all three configurations (1.7 cm, Fig 1). Sterile saline wipes (Hygea, PDI Inc., NY) were applied on the skin to ensure the electrode contact area was sufficiently moist prior to performing impedance measurements. The recording software of the EIM HEA also displays a contact check prior to each measurement, and also immediately plots the resistance and reactance across the range of frequencies. Three EIM measurements were performed for each subject at rest within 2–3 minutes and the array sensor position was maintained at the same location over the skin throughout data collection. The software display was used to visually inspect the data to ensure the data of the three trials were consistent and smooth. Subcutaneous fat or SFL thickness was separately measured by ultrasound (S-Cath, Sonosite Inc., WA) with a linear probe (6–13 MHz) placed on the center of muscle belly, approximately at the same location where the EIM data was collected. The probe was placed on the relaxed muscle, perpendicular to the direction of the muscle fibers. Ultrasound gel was applied on the probe to ensure good contact between the ultrasound probe and the skin, as well as to avoid applying any excess pressure. The ultrasound images were taken by an experienced operator. Once a quality ultrasound picture was captured, electronic calipers within the Sonosite software were used to measure the SFL thickness (Fig 2). The mean SFL thickness value from three satisfactory images was used for analysis.

### Data analysis and statistics

The resistance (R), reactance (X) and phase (θ) (mean ± standard deviation) were analyzed and reported. This data was used to generate plots describing the relation between EIM parameters (X, R, θ) and applied frequency. The data obtained at 50 kHz was selected to be used in a correlation analysis with subcutaneous fat thickness. This frequency was chosen as it is the most commonly utilized frequency of EIM and is considered to be in the optimal range of EIM responses. The ratio of 50 kHz to 100 kHz of phase was calculated and its correlation with SFL thickness was also tested. Pearson correlation was used to determine the coefficient of determination between the EIM parameters and SFL thickness. All analyses were performed using MATLAB (Mathworks, Natick MA). Significance level was determined as p < 0.05 for all the statistical analyses.

## Results

### Subcutaneous fat thickness

**Fig 3:**
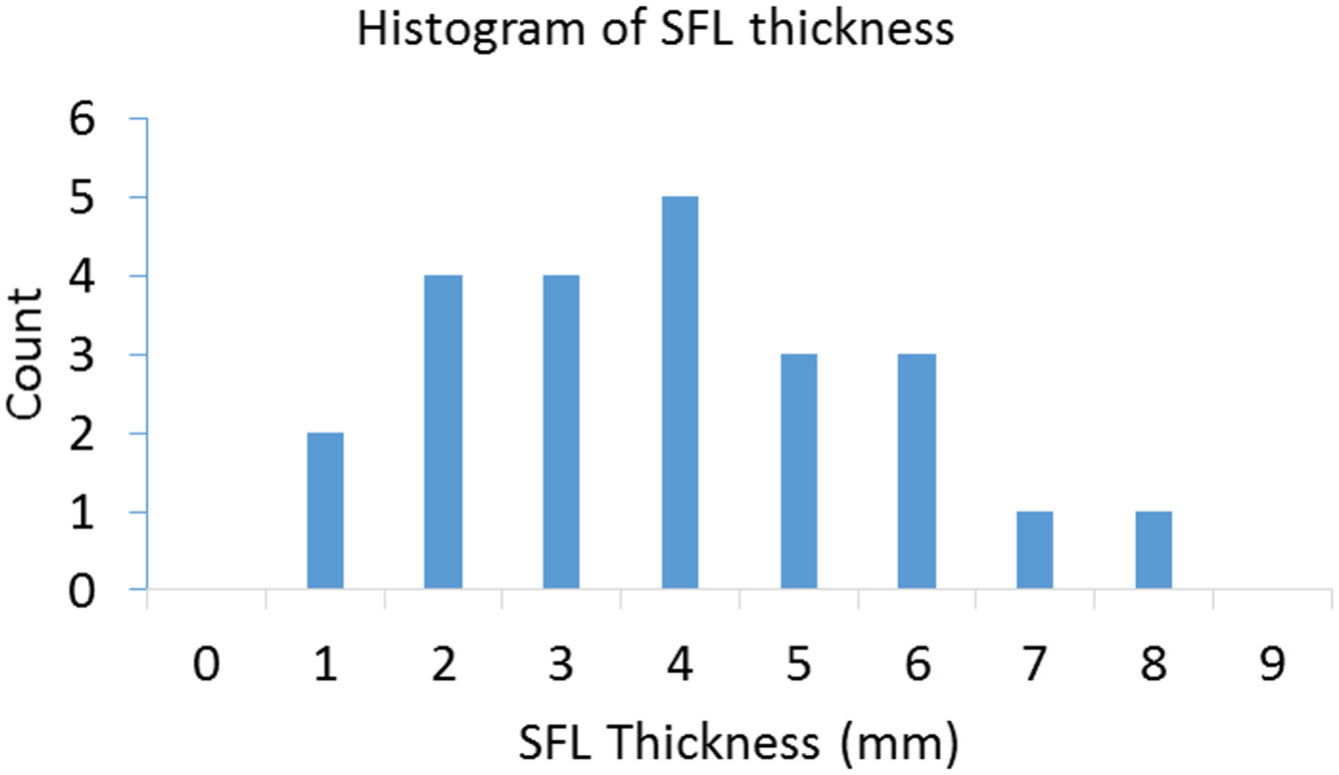
Histogram showing the distribution of measured SFL thickness overlying the biceps in the recruited 23 healthy subjects.

Fig 3 shows the distribution of the SFL thickness from all the subjects, which had a range from 1.7 mm to 8.47 mm. The mean value was 4.5 mm with a standard deviation of 1.8 mm. There was no significant difference of SFL thickness between males and females (p = 0.07).

### Effect of SFL on EIM at 50 kHz

**Fig 4:**
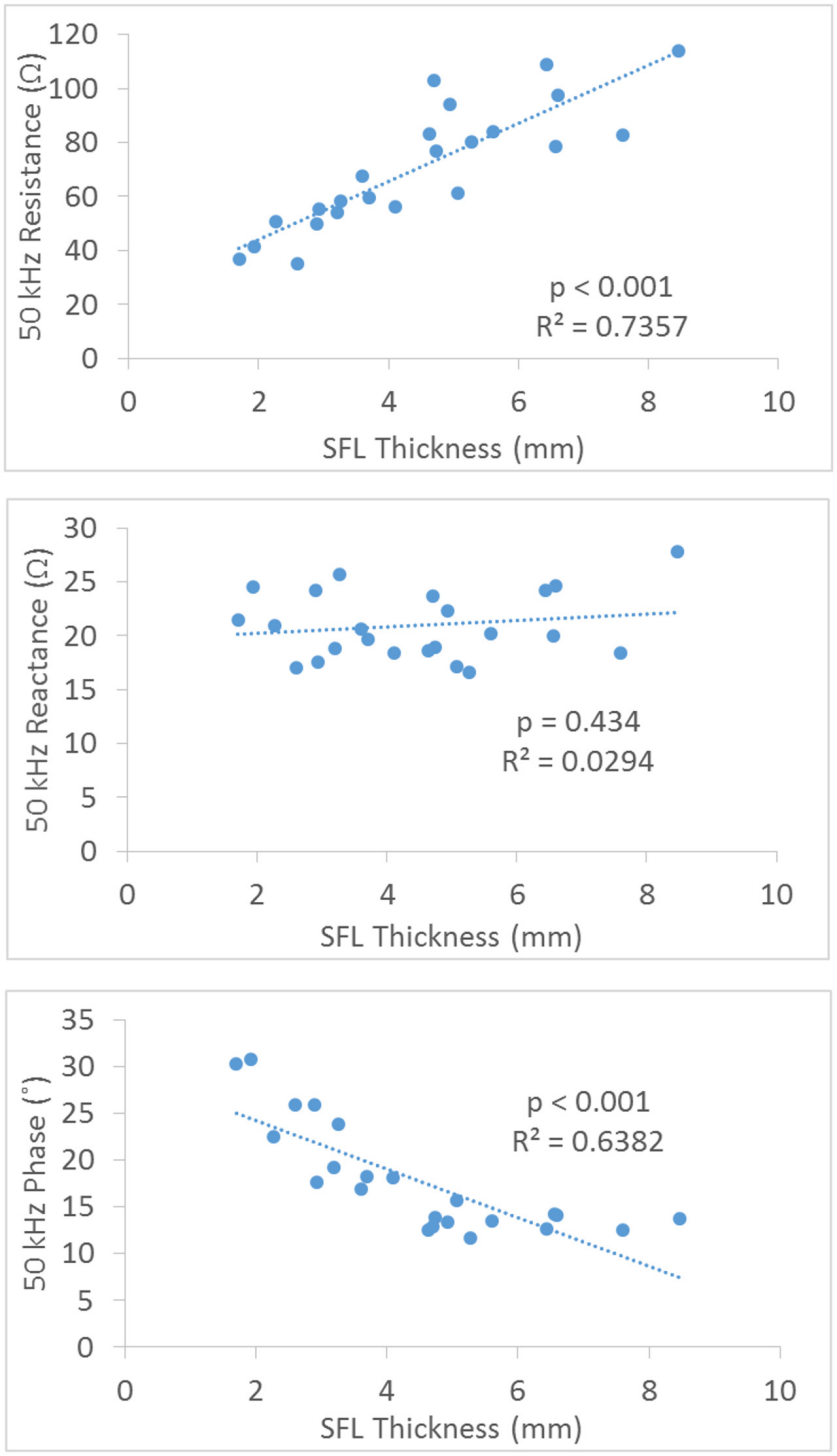
Correlation plots for the resistance, reactance and phase at 50kHz EIM versus SFL thickness using a transverse configuration of electrodes.

Fig 4 shows the correlation between SFL thickness and the EIM parameters at 50 kHz frequency from the narrow transverse configuration of the current electrodes. Only the resistance and phase showed significant correlation with SFL (R^2^ = 0.7357 and 0.6382, p<0.001), and there was no significant relationship between the reactance and SFL (R^2^ = 0.0294, p = 0.434).

**Fig 5:**
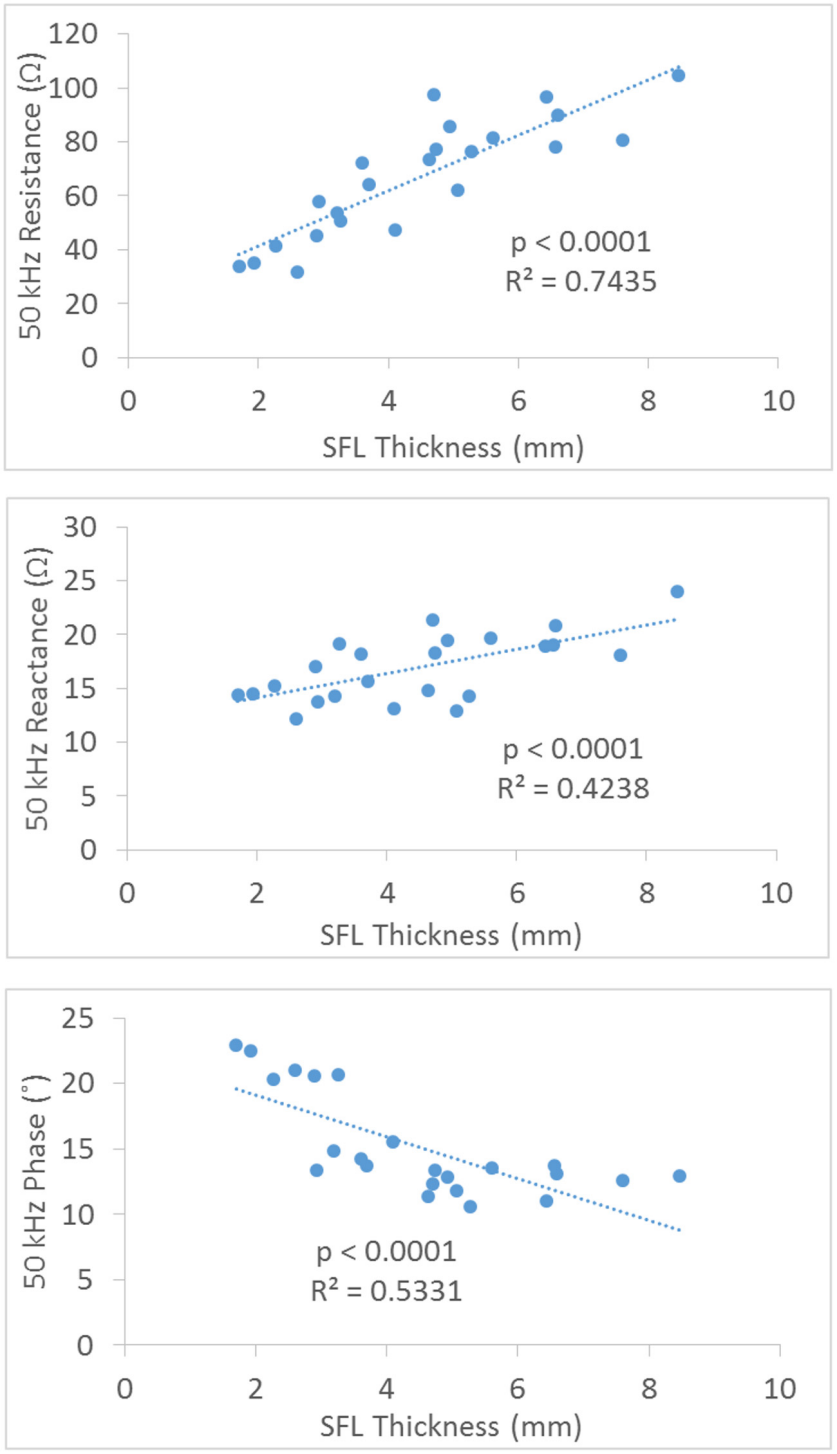
Correlation plots for the resistance, reactance and phase at 50kHz EIM versus SFL thickness using a narrow longitudinal configuration of electrodes.

**Fig 6:**
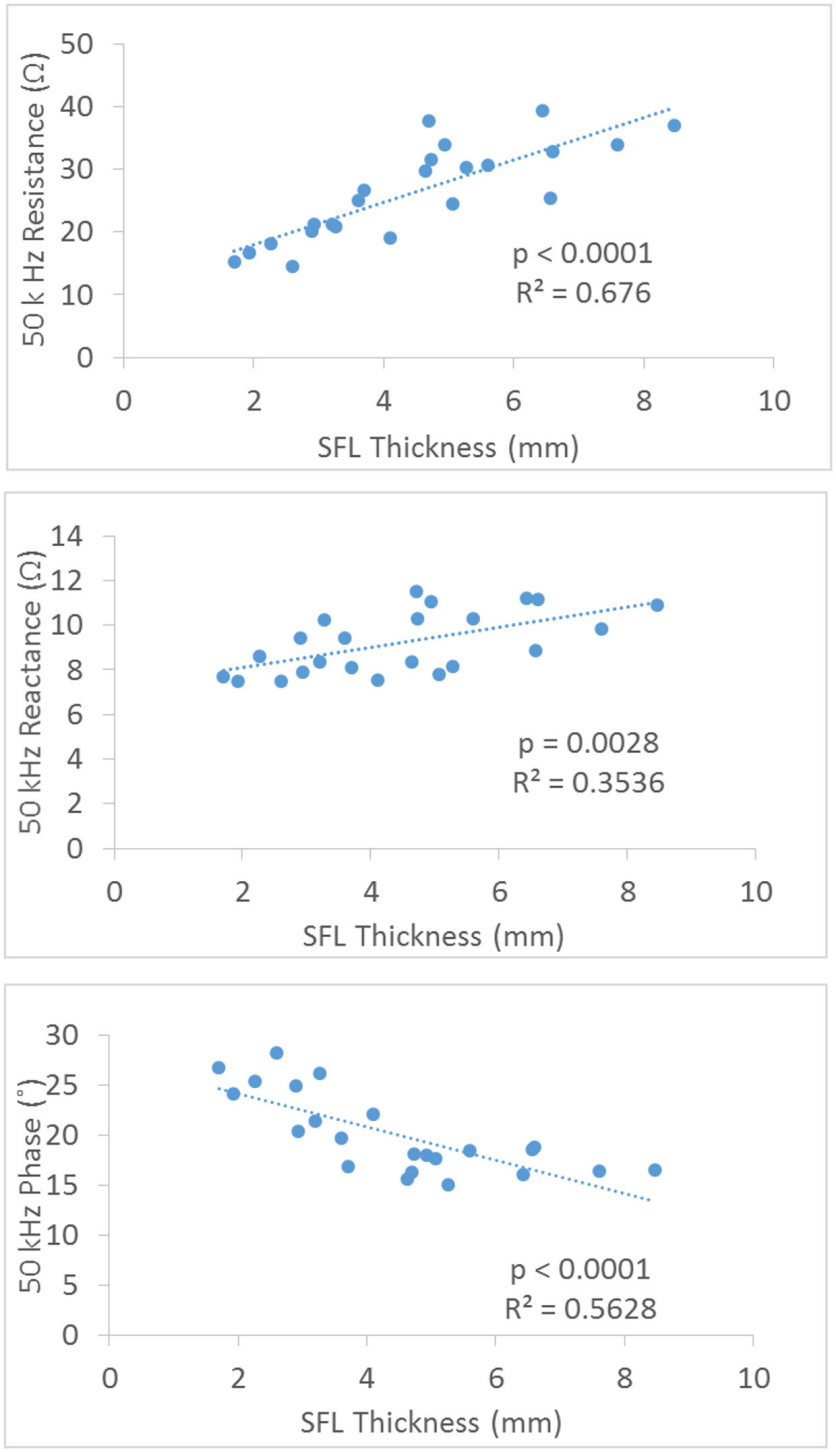
Correlation plots for the resistance, reactance and phase at 50kHz EIM versus SFL thickness using a wide longitudinal configuration of electrodes.

Fig 5 shows the correlation between SFL thickness and the EIM parameters at 50 kHz frequency from the narrow longitudinal current configuration. There were significant positive relationships between the resistance, reactance and SFL thickness (with coefficient (R^2^) values of 0.7345 and 0.4238 respectively, p<0.0001 for both), and a significant negative correlation between the phase and SFL thickness with coefficient (R^2^) value of 0.5331 (p<0.0001). Fig 6 shows the correlation between SFL thickness and the EIM parameters at 50 kHz frequency from the wide longitudinal current configuration. The results were similar to the narrow longitudinal data but the increased distance reduced the correlation relationship for both resistance and reactance.

### Multi-frequency analysis

**Fig 7:**
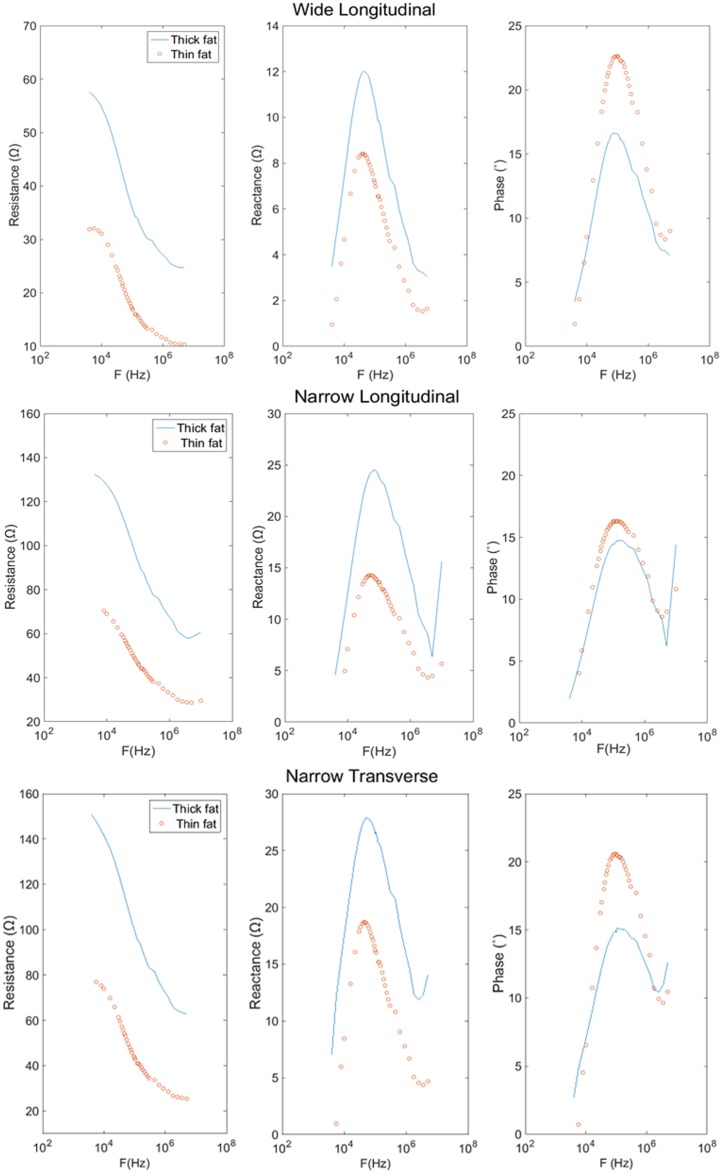
Resistance, reactance and phase vs. logarithm of frequency for biceps brachii of a 34 year old female (solid line) and a 35 year old male (dotted line) using different electrode configurations.

**Fig 8:**
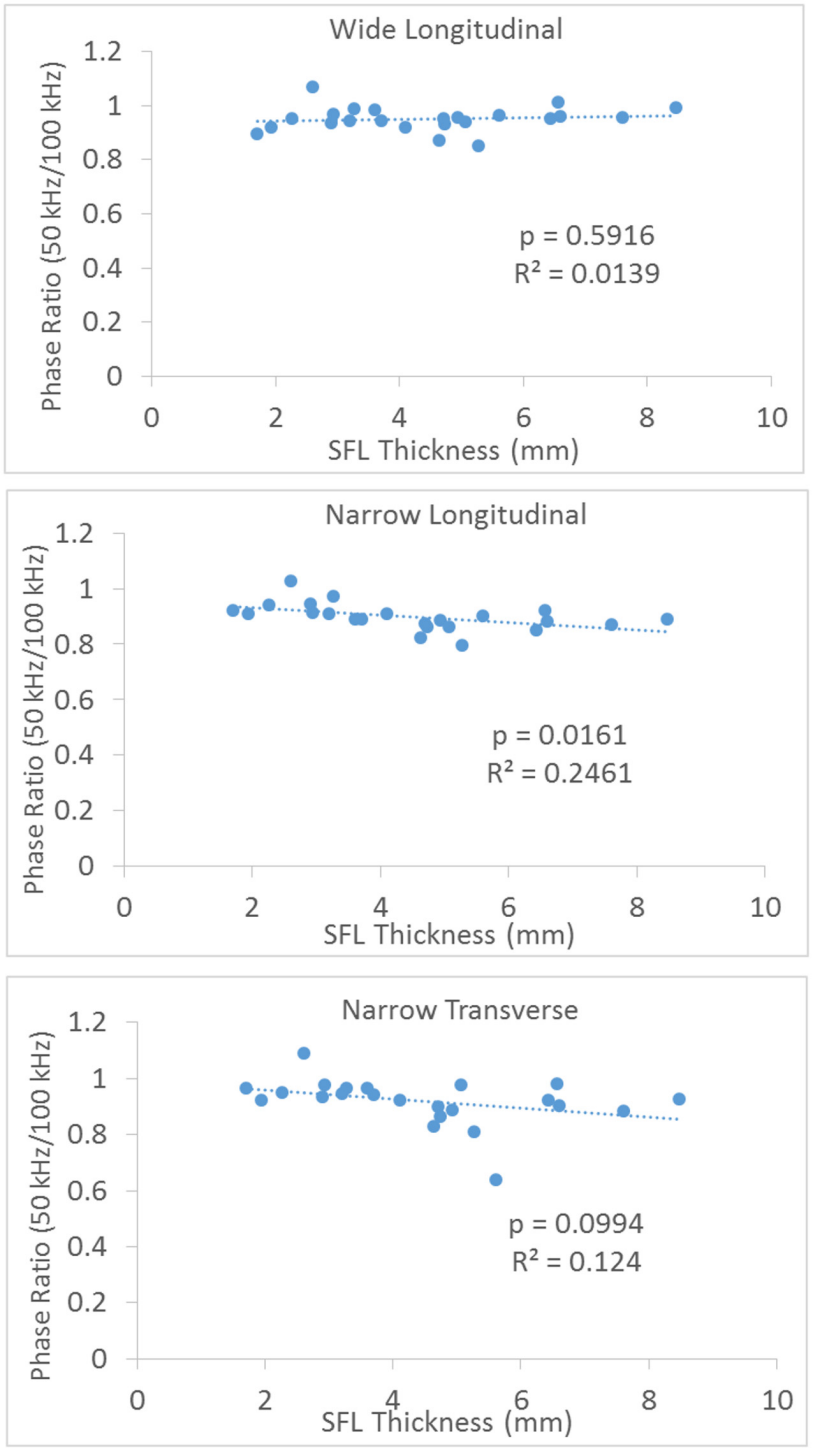
Correlation of 50 kHz/100 kHz phase ratio with SFL thickness.

Fig 7 shows a typical relationship between the resistance, reactance, and the emitting frequency for a 34 year old female subject with SFL thickness of 8.3 mm and a 35 year old male subject with SFL thickness of 4.1 mm. For all three configurations, the larger SFL thickness increased the resistance (across all the frequencies), increased the peak reactance and decreased the peak phase. With an increase of the frequency, the resistance decreased, whereas the reactance and phase demonstrated a peak value and then decreased at higher frequencies. Among the configurations, we observed that the wider electrodes reduced the measured resistance and reactance. The SFL thickness showed no significant impact on the 50kHz/100kHz ratio of phase for the wide longitudinal and narrow transverse electrode configurations (Fig 8).

## Discussion

Changes in impedance can arise from changes in the nature of the conducting medium (such as the skin, subcutaneous fat layer, muscle and other connective tissues) or from changes in geometric factors [17]. There have been varying observations on how much the fat might influence the EIM data. For example, Garmirian et al. argued that generally the fat effects would be minor except in perhaps obese subjects [18]. Baumgartner et al. specifically found bioimpedance differences in obese subjects with morphometry of different tissue compartments under MRI [19]. In this study, correlations between localized EIM parameters and SFL thickness were investigated using different electrode configurations and by applying current at a range of frequencies.

The average thickness of SFL in this study is 0.45 ± 0.18 cm (n = 23), similar to the findings of Kortman et al. that reported SFL thickness of 0.44 ± 0.04 cm (n = 16) at upper limb from healthy subjects with age from 19 to 50 years [9]. In addition, we did not find a significant difference of SFL thickness between men and women. Kortman et al. also found no gender effects of SFL thickness on EIM of upper limb [9]. It appears that the correlation between the EIM parameters and SFL thickness is closely related to the distance and direction between the two current electrodes.

For all configurations, the resistance showed a strong correlation with SFL thickness. This finding is consistent to previous findings in the gastrocnemius muscle of the lower limb [13]. All tissues underneath the voltage electrodes could theoretically contribute to the impedance signature, including subcutaneous fat and bone. The inherent resistivity of muscle is an order of magnitude lower than that of fat and two orders of magnitude lower than that of bone [20]. Thus the electrical current tends to travel through the muscle. However, as suggested by Sung et al., when currents flow through tissues under the voltage-measuring electrodes, a considerable proportion of current will be going through the fat at the points closest to where the voltage is measured [13]. Therefore, straightforward comparison of linear EIM and localized EIM need to be given extra attention regarding the different electrode arrangements and the muscles evaluated. Jafarpoor et al. used a finite element model to evaluate the effects of size and conductivity of muscle and subcutaneous fat thickness on the EIM and the results showed the resistance increased 375% when there was a quadrupling of fat thickness [15]. Our high resistance data from healthy subjects can also justify relatively low resistance in animal EIM studies since the animal impedance was directly measured on the muscle tissue [21].

The reactance measured from the transverse configuration may reveal the effect of electrode direction on reducing the impact of the subcutaneous fat. The effect of the direction of the current on reactance is related to the anisotropic properties of the muscle which has been evaluated by Chin et al. [2] and Garmirian et al. [18]. Skeletal muscle is electrically anisotropic and current flows more easily in the longitudinal direction (parallel to the muscle fibers) than in the transverse direction. Since subcutaneous fat is considered as an isotropic tissue [7], the transverse electrode configuration in this study likely led to a much stronger capacitive effect on reactance from muscle tissue and therefore making the proportion of SFL effect relatively smaller compared to the longitudinal configuration (Fig 4). For the distance factor, Sung et al., using a hand-held array with longitudinal current electrodes 6 cm apart, found the SFL thickness only had an impact on the resistance and phase, but not the reactance of the gastrocnemius [13]. This finding of the relatively small influence of fat on reactance is in line with the modeling study of Jafapoor et al, which showed that when the distance between current electrodes was large (10 cm) the reactance only slightly changed with significant increase of fat [15]. In an in-vitro study of bovine skeletal muscles, Tarulli et al. also found that EIM measurements are dependent on the exact size of both the current and voltage electrodes, as well as the distance between the electrodes [22].

Phase represents the way cell membranes shift the timing of oscillations in voltage relative to those of an applied alternating current. It was previously used as a major outcome measure of EIM since it is considered to be less affected by the architecture and shape of the muscle as compared with resistance or reactance [10]. Tarulli et al. found that the SFL thickness does not contribute substantially to the phase measured by linear EIM on quadriceps [14]. Our results showed a negative correlation of SFL thickness to localized phase data. In the study of Tarulli et al., the current electrodes were placed at a far distance from the measuring voltage electrodes. Thus relatively little current can flow along the subcutaneous fat layer because fat has substantially higher resistivity and lower cross-sectional area than the muscle [20]. In addition, in linear EIM the voltage measurement along the length of the limb makes the proportion of instrument impedance relatively large and the contribution from SFL relatively small [10]. However, in the current study, the hand-held array only allows shorter distance between the electrodes, which may be the cause of the strong correlation between phase and SFL thickness. A similar finding was also reported by Sung and colleagues for the median gastrocnemius muscle [13].

Early studies in tissue electrical properties laid the groundwork for bioimpedance analysis. Valdiosera et al. determined the passive electrical properties of cells as well as the equivalent circuit for current flow in the skeletal muscle of frogs [23]. Cole and Cole interpreted impedance measurements in biological tissues by utilizing simple circuit representations of the body and visualizing plots of reactance vs. resistance, known today as “Cole-Cole plots” [24]. From those classic models and plots, we know that the impedance of a membrane depends on the frequency of the applied current, and consequently so does the measured resistance, reactance, and phase. Changing the frequency of the injected current shifts the relative weights of resistive (fluid) and reactive (membranes) contributions on the impedance, and indeed at very high frequencies the cell membranes make nearly no contribution [4] [25]. With an increase of current frequency (Fig 7), the resistance is decreased as demonstrated in the current study. In addition, we used a 50kHz/100kHz ratio of parameters and demonstrated a large reduction of the subcutaneous fat effect on phase (Fig 8). These findings support that using multi-frequency EIM and normalization could be useful in reducing the fat effects [7]. Although the 50kHz/100kHz ratio was used in this study, it is not necessarily the optimal pair to minimize the effect of the subcutaneous fat. The selection of 50 kHz was primarily because it is most frequently used for muscle examination and commercial bioimpedance devices are usually readily available at this frequency. We noted that when examining muscles in DMD subjects, Schwartz et al. found that the 50kHz/200kHz ratio was most appropriate among the tested frequency pairs to reduce the correlation of the subcutaneous fat thickness [7].

The EIM array used in this study had smaller voltage electrodes (0.3 cm wide, 1.2 cm long) compared with those (0.75 cm wide, 2.5 cm long) used in the study by Sung et al. [13]. Reducing the size of the electrodes also increases the likelihood of interfering capacitive and resistive effects at the electrode–tissue interface [22]. Therefore, the effect of electrode size on EIM may need further investigation. In addition, this study only measured the subcutaneous fat thickness in vivo and correlated it with EIM parameters, whereas the intramuscular fat in this group of healthy individuals was not quantified. Diseased muscles such as hemiparetic skeletal muscles after stroke may have increased intramuscular fat [26], which will also potentially affect the EIM parameters. It is possible that greater subcutaneous fat might predict greater intramuscular fat. Imaging techniques, such as quantitative ultrasound for measuring composition [6], might be a useful way to further explain the electrical impedance changes.

In summary, our results indicate that for the longitudinal current electrode configuration, the wide distance can reduce the effect of SFL thickness on the reactance of the biceps brachii muscle. For the transverse current electrode configuration, although a significant correlation still remained for resistance and phase, there was no significant correlation between reactance and SFL thickness. A 50kHz/100kHz ratio of the phase also reduced the effect of SFL thickness. These findings indicate that the impact of the SFL on EIM can be reduced by an appropriate selection of the distance and direction of the current electrodes as well as the multi-frequency phase ratio. Therefore, when using hand-held localized arrays to perform EIM, these settings or parameters can be optimized to reduce the effect of subcutaneous fat variation, which can confound the EIM data interpretation. However, for human clinical studies, further assessment is required to determine whether these settings or parameters are clinically meaningful with strong correlation to clinical status in different diseases.
